# Characterization of primary and secondary cognitive impairment in schizophrenia

**DOI:** 10.1192/j.eurpsy.2026.10346

**Published:** 2026-07-31

**Authors:** A. Vita, Gabriele Nibbio, Jacopo Lisoni, Giacomo Deste, Stefano Barlati

**Affiliations:** Department of Clinical and Experimental Sciences, University of Brescia, Brescia, Italy

## Abstract

**Abstract:**

Cognitive impairment associated with schizophrenia (CIAS) affects over 80% of patients and represents a core feature of the disorder, demonstrating greater predictive value for functional outcomes, more than positive symptoms. Understanding the conceptual framework distinguishing “primary” from “secondary” cognitive impairment is essential for targeted therapeutic interventions.

Primary cognitive impairment reflects the intrinsic neurodevelopmental pathology of schizophrenia, manifesting across multiple domains including processing speed, attention, working memory, verbal learning, executive functions, and social cognition. These deficits typically predate psychosis onset, are observed in unaffected first-degree relatives, and remain relatively stable throughout the illness course regardless of symptom remission, with global cognitive performance averaging one standard deviation below general population.

Secondary cognitive impairment results from modifiable extrinsic factors that further compromise cognitive performance beyond the primary deficit. Key contributors include anticholinergic medication burden, first-generation antipsychotic side effects, benzodiazepine use, metabolic syndrome, sleep disturbances, sedentary lifestyle, substance abuse (particularly cannabis), and social deprivation.

Distinguishing primary from secondary cognitive impairment has critical therapeutic implications. While primary deficits require evidence-based interventions, such as cognitive remediation and optimized pharmacological treatment, secondary impairments demand systematic identification and mitigation of contributing factors including medication rationalization, metabolic management, aerobic exercise programs, substance abuse treatment, and social and work inclusion programs.

This conceptual framework provides clinicians and researchers with a structured approach to assessment and personalized intervention strategies, ultimately improving functional recovery in schizophrenia.

**Disclosure of Interest:**

None Declared

